# Classic Pro‐Choice Thought Experiments and African Communitarianism

**DOI:** 10.1111/bioe.70016

**Published:** 2025-07-16

**Authors:** Kirk Lougheed

**Affiliations:** ^1^ LCC International University Klaipeda Klaipėdos Lithuania; ^2^ University of Pretoria Pretoria Gauteng South Africa

**Keywords:** abortion, African bioethics, cross‐cultural bioethics, pro‐choice thought experiments

## Abstract

I analyse two classic pro‐choice thought experiments in the Anglo‐American philosophical literature in Thomson's Violinist Case and Tooley's Kitten Serum Case, in light of two prominent African normative theories. Though each of these cases is designed to generate pro‐choice intuitions, I suggest they do not do so nearly as clearly when African normative theories are in view. Furthermore, even where they might yield a pro‐choice verdict, they do so for very different reasons. That African ethics, which is often labelled communitarian, differs from what one typically finds in the Anglo‐American normative tradition is hardly a new insight. However, that these differences might undermine the universality of pro‐choice thought experiments about abortion in Anglo‐American bioethics has yet to receive significant attention.

## 1 Introduction

African philosophy in the narrow sense of being conducted by professional philosophers employed by universities largely emerged in sub‐Saharan Africa in the 1960s, though not exclusively. Indigenous black philosophers have written philosophically about a wide variety of ideas that are important in different African cultures. Given the political and economic instability that exists throughout much of the continent, it is of little surprise that moral and political philosophy have occupied much of the focus.^1^ When referring to an idea as ‘African’, I just mean that it has been salient to indigenous black Africans in the sub‐Saharan region for an extended period of time. This is not to imply that the idea is exclusive to the continent. For example, it is reasonable to say that baseball is American, even though it is played in Japan [1]. For the most part, the contemporary philosophical literature is written in English, though the impact of translating certain concepts from their indigenous languages into English is sometimes noted.

Within African moral philosophy, debates in normative ethics often take centre stage. One important strain of normative theories focuses on the concept of personhood, which is a normative term in this tradition, referring to the moral achievement of an individual [2, 3]. One interpretation says that the goal of morality is to develop one's personhood, which is done by exercising other‐regarding virtues which can necessarily only be done in the context of community [4]. Since normative personhood is one of the most important concepts in African philosophy, it is unsurprising that there are numerous interpretations of this concept and much debate over how it should be applied to different moral questions [5, 6, 7].

Another set of normative theories focuses on the value of communal harmony [8, 9]. Many suggest that communal harmony is the highest good and therefore needs to be the sole goal of any moral decision‐making. Such theories are fairly construed as teleological approaches to communal harmony [10, 11, 12]. A different theory that also focuses on communal harmony is deontological in saying that it is a person's capacity to relate harmoniously with others that is of value [1]. People are owed respect in virtue of possessing this capacity.

Finally, what is reasonably called a religious ethic draws on the key claim in African Traditional Religion which says that literally everything is imbued with an imperceptible energy known as life force or vital force [13, 14, 15]. The goal of morality on this view is to strengthen the force in oneself and in others. There are recent attempts that explore the extent to which such an ethic can be successfully naturalised [16].

Given that African normative ethics is a thriving field that sometimes leads to much discussion of various topics in applied ethics, it is surprising that comparatively little has been written on the topic of abortion in African philosophy. Recent exceptions include the American philosopher (long‐time based in South Africa) Thaddeus Metz, who says his relational theory entails that early‐stage abortions are permissible, while later‐stage ones are typically impermissible [1, p. 183]. The South African philosopher Motsamai Molefe believes his interpretation of an ethic based on personhood entails that *all* types of abortion are impermissible except those required to save the life of the mother [17]. Earlier work includes the ideas of Cameroonian bioethicist Godfrey B. Tangwa, who explains that for the Nso, abortion is taboo [18].^2^ The Congolese theologian Bénézet Bujo, in explicating the implications of what he calls a traditional African worldview, explains that abortion is impermissible because the embryo and/or foetus are fully emersed in the community of living and dead, having the status of ‘not‐yet born’ [19]. Indeed, traditional African worldviews are usually considered to highly value procreation [20]. Finally, there is a handful of other work on abortion that is less imbedded within a systematic framework, but that also tends to be pro‐life.^3^

My aim is to focus on what two different African normative theories have to say about abortion from the perspective of two classical pro‐choice thought experiments in the Anglo‐American literature. As far as I can tell, such a project has not been undertaken in any detail to date. The normative theories I appeal to are attractive choices for at least three reasons. First, they are two of the most *systematic* theories on offer in the African literature, having been explicated in full‐length books. Second, they are *representative* of two significant branches of African normative ethics. Third, they are *secular* and therefore consistent with metaphysical naturalism. This increases the likelihood that they will be palatable to global audiences beyond the African continent. It is my hope that this approach will not only expand the current African philosophical literature on abortion, but it will help pave the way for future cross‐cultural dialogue on important topics in bioethics.

The Anglo‐American philosophical literature on abortion is full of hypothetical cases and thought experiments intended to generate intuitions either for or against the permissibility of abortion. Indeed, one sometimes gets the sense that the cases are more important than debates about specific moral principles or moral intuitions generated apart from them. In what follows, I analyse two important classic pro‐choice thought experiments in the Anglo‐American philosophical literature in Judish Jarvis Thomson's Violinist Case and Michael Tooley's Kitten Serum Case in light of two different African normative theories. Though each of these cases are designed to generate pro‐choice intuitions, adopting different African normative theories which tend to be highly communitarian in nature, casts doubt on the intuitions the cases are typically thought to generated. Furthermore, even where they might yield the desired pro‐choice verdict, they do so for very different reasons than those Thomson or Tooley appear to have in mind. That African communitarian ethics differs from what one typically finds in the Anglo‐American normative tradition is hardly a new insight, but this observation has yet to be systematically applied to important thought experiments on abortion. Doing so may well show that the purported lessons of these thought experiments do not generalise across cultures as previously implicitly supposed. All of this points to the fact that despite the appeal of thought experiments, it might turn out to be difficult from a methodological perspective to use them if one is seeking to make truly cross‐cultural and universal or objective claims.

## 2 Two African Normative Theories

In what follows, I briefly outline the two African normative theories that I will use to evaluate the two classic Anglo‐American pro‐choice thought experiments. Though I described three main strains of normative theories above, there are dozens of variations within each of them, such that it would be impossible to cover them all here. I therefore choose two influential theories, one that focuses on normative personhood and the other that emphasises communal harmony. Not only are these influential, but they both offer explicit theories of right action, and their proponents have used them to address the permissibility of abortion.

### 2.1 Normative Personhood

The first theory I will use to examine the thought experiments is an account of normative personhood developed by Motsamai Molefe (2019, 2020). He is one of the few philosophers to develop such an account and explicitly apply it to abortion (see Section 3.1). Molefe's normative theory encompasses a number of features, including the idea that the goal of morality is character‐based and perfectionist egoist, grounds a moral status, and offers a clear theory of right action. In explicating his theory, I will primarily work from his two books, *An African Philosophy of Personhood, Morality, and Politics* (2019), and *An African Ethics of Personhood and Bioethics: A Reflection on Abortion and Euthanasia* (2020).

First, Molefe's theory is character‐based and perfectionist egoist because the goal of morality is to develop oneself by developing one's character or virtues [4, p. 10]. The character traits that matter are other‐regarding virtues, and so need to be developed in the context of community. Indeed, Molefe suggests that the overarching other‐regarding virtue at which to aim is sympathy. He writes that

[A]ll other‐regarding virtues associated with personhood are generated from and expressive of the foundational virtue of sympathy. Thus, instead of explaining a virtuous human being in terms of a grab‐bag list of virtues like kindness, compassion, friendliness and generosity […] we can now account for moral perfection in terms simply of sympathy. In this interpretation of matters, it follows that to possess personhood just is to be sympathetic; and the goal of morality is to develop this moral capacity [4, p. 55].

Being sympathetic to someone encompasses having kindness, compassion, friendliness, and generosity [4]. It is also a moral sense that indicates ‘concern’ for another [4]. Molefe writes that ‘it is generally true that this capacity to connect, to be responsive to the needs of others and to emphasise the importance of social responsibility can best be accounted for by appeal to the primacy of sympathy in the discourse of personhood’ [4, pp. 56–57]. In some indigenous languages (e.g., Ngunni and Sotho) sympathy can literally be translated as ‘to hear’ [4, p. 57]. Though ‘listening’ is an innate capacity, it can be developed (in the moral sense) over time. Thus, ‘[t]he idea of sympathy thus refers to the human ability or capacity to be sensitive and responsive (to ‘hear’ and ‘listen’) to the condition of others’ [4, pp. 57–58].

Second, with respect to moral value, Molefe believes that it is the *capacity* for sympathy that grounds the value of persons [4, p. 88]. It is this capacity that explains why humans have a dignity. Finally, third, though Molefe says that though ‘[m]orality is captured primarily in terms of character development, […] character development requires the development of a moral disposition of habits’ [4, p. 63]. He cites the work of Metz approvingly here, someone who has spent his career developing an African theory of right action [24]. Molefe says practising the virtue of sympathy will help it to become a habit that steers right action. This means that ‘[r]ight actions tend to strengthen our social relationships, which in turn help the agent realise her true nature and develop a virtuous character’ [24].

### 2.2 Communal Harmony

Though the highly communitarian nature of almost all branches of African normative ethics has been frequently observed, some theorists claim that communal harmony itself should be the focus of morality. Thaddeus Metz has been defending a deontological interpretation of this type of ethic for a number of years, culminating in one of the most systematic African normative theories on offer in his book, *A Relational Moral Theory: African Ethics in and beyond the Continent* (2022) [1]. For Metz, human beings are valuable inasmuch as they have the capacity for ‘friendliness’, a technical term for something like the capacity to love and be loved. It is this capacity that grounds human dignity and hence the requirement for honorific treatment. His shift to deontology is motivated by his doubts that teleological approaches to harmony are able to provide grounds for human rights (or dignity) [1]. Metz's theory ‘is not that communal relationship itself has a moral status, nor that only those who are in such a relationship have one, but rather that those who in principle could relate in that way have a moral status’ [2, p. 106]. He explicates friendliness in terms of two concepts, identification and solidarity.

Metz explains that the cognition of identification implies thinking about oneself as part of a group with others. Others are part of your group, not separate entities [1, p. 94]. The emotions involved in identification will be feelings of security and a sense of belonging in another's presence. You will be embarrassed or proud of the actions of others. The volition of identification is about helping others to achieve their goals, while framing your own goals within the group. The motivation for helping others is not primarily a function of how well doing so would help you achieve your own goals [1, p. 95]. The opposite of identification is fostering division, while neither identifying with others nor being divided with them is probably alienation [1].

Solidarity with others is first and foremost about acts of service. Its cognition involves having empathy for others and so ‘knowing what moves [the other…] person and more generally what makes him tick, even if he does not fully recognize it’ [1, p. 96]. This type of empathy is needed to develop emotional solidarity. With respect to the volition of solidarity, it implies taking concrete actions to help improve the lot of others [24]. Such solidarity is motivated ‘by the prior conditions of roughly empathetic cognition and sympathetic emotion’ [1]. ‘Ill will’ is the opposite of solidarity, where neither solidarity nor ill will is probably indifference [1, p. 97].

It is the capacity for identifying with others and exhibiting solidarity with them that grounds moral value. Accordingly, his theory can be captured by the following principles:

- An act is right if and only if it respects individuals in virtue of their capacity to be party to harmonious ways of relating.
- An act is wrong insofar as it degrades those with the capability of relating communally as subjects or objects.
- An action is permissible if it treats beings as special in accordance with their ability to be friendly or to be befriended.
- An action is impermissible to the extent that it disrespects beings with the ability to be part of relationships of identity and solidarity [1, p. 110].

In sum, an act is right inasmuch as it respects a person in light of their capacity for friendliness, where those acts that degrade this capacity are impermissible.

## 3 Thomson's Violinist Case

What is undoubtably the most cited hypothetical case in the contemporary Anglo‐American literature on abortion is Judith Jarvis Thomson's Violinist Case. Here is the case in full:

You wake up in the morning and find yourself back to back in bed with an unconscious violinist. A famous unconscious violinist. He has been found to have a fatal kidney ailment, and the Society of Music Lovers has canvassed all the available medical records and found that you alone have the right blood type to help. They have therefore kidnapped you, and last night the violinist's circulatory system was plugged into yours, so that your kidneys can be used to extract poisons from his blood as well as your own. The director of the hospital now tells you, ‘Look, we're sorry the Society of Music Lovers did this to you‐we would never have permitted it if we had known. But still, they did it, and the violinist now is plugged into you. To unplug you would be to kill him. But never mind, it's only for nine months. By then he will have recovered from his ailment, and can safely be unplugged from you’ [25].

Thomson's point is that even if the foetus is a person, there are cases where it is permissible for the woman to *detach* herself from it. There are cases where a woman has not consented to the foetus using her body's resources to stay alive. The most obvious of these cases are those of nonconsensual intercourse, though Thomson extends her argument to apply to cases where contraception is used [25, pp. 58–59]. According to Thomson, even on the assumption that the foetus is a person, her argument establishes that in certain situations the foetus has no right to use the mother's bodily resources, and it is in those cases that abortion is permissible.^4^

### 3.1 The Violinist Case and Normative Personhood

In this subsection, I consider how the Violinist Case would be evaluated from the perspective of Molefe's conception of personhood. The focus of the response is immediately different given that sympathy is an overarching *other‐regarding* virtue. What is it morally permissible for the person who finds themselves attached to the violinist to do? According to personhood as sympathy, the question they must ask themselves is whether unplugging is sympathetic or unsympathetic. I suggest that a plain reading of sympathy implies it would probably be unsympathetic for the person to unplug themselves from the violinist. Since it would be unsympathetic to the violinist to unplug oneself, it is immediately obvious that autonomy is valued significantly less on personhood than is usually the case in the Anglo‐American tradition. This is consistent with much less consideration being given to the value of autonomy throughout the African philosophical tradition [18, 27].

Expanding further, the person who is connected to the violinist needs to ask themselves how their decision will affect not just themselves and the violinist, but how their decision will affect the entire community.^5^ Part of what complicates the case when considering it from the perspective of personhood is that the Society of Music Lovers ought to consider whether plugging you into the violinist without considering your needs and discussing the matter with you in the first place is permissible.^6^ It is very doubtful that plugging you into the violinist is sympathetic. Thus, in a society that prioritises developing one's personhood as the capacity for sympathy, the sort of situation described by Thomson is unlikely to come about in the first place.

However, a counterpoint to this is that sympathy *for the violinist* does indeed prescribe plugging you into the violinist.^7^ Sympathy for the violinist (and the community that appreciates their music), means working to save them. This is a specific instance of a more general problem for virtue theories of which Molefe's theory may well succumb. The difficulty is the inability to prescribe right action in light of virtues at work in multiple people. In other words, how does personhood as sympathy say we should choose between sympathy to the violinist and sympathy to the innocent person that could save them? Where Molefe's account differs from other Anglo‐American virtue theory is that he believes humans are valuable inasmuch as they have the *capacity* for sympathy. This capacity is what grounds the dignity and respect of persons. Innocents cannot be killed against their will to save others or for the greater good because they have a dignity. Notice that even if I am wrong about how Molefe can deal with this objection, Thomson still cannot generate the pro‐choice intuition.

Though Thomson's case may generate pro‐choice intuitions in a Western or Anglo‐American or Anglo‐American context, it fails to do so from the perspective of personhood as sympathy. If a decision is absolutely forced, and you must decide whether to unplug using an ethic of personhood as sympathy, it is impermissible for you to disconnect. This is especially so if remaining plugged in would benefit the relevant moral community of which you are a part. If the Society of Music Lovers comprises (much of) the relevant community, then this is an even stronger reason for you to stay connected. At this juncture, the reader should remember that I am *not* defending this as the correct response to the Violinist Case. Instead, I am merely observing that on a normative theory which says the goal of morality is to develop one's personhood, where this is understood as exercising sympathy, the intuitions that Thomson are driving at cannot be upheld.

Here is a potential objection to this line of reasoning: The African moral tradition often says ‘family first’ or that ‘charity starts in the home’ with special duties owed first and foremost to one's immediate family, follow by extended family and close friends [1, p. 117]. What needs to be considered, then, is not only how the Society of Music Lovers and wider society will react to losing the violinist, but also how being forcibly plugged in will affect your immediate family and friends. They potentially lose out on an important member of their community for 9 months.

Consider that personhood as sympathy not only implies sympathy for the members of the Society of Music Lovers, it means you also must be sympathetic to your immediate family and friends. In fact, they ought to be a special object of your sympathy. Molefe is explicit that personhood entails special obligations. He writes that 'a *person*, all things being equal, ought to start the dispensing of their moral duties with their special relations […] the emphasis [is] on other‐centred partiality’ [17, p. 86, 28]. For Molefe, personhood implies partiality. He believes that his theory of personhood as sympathy is agent‐centred in that the agent must be focused on perfecting her humanity. This means ‘the agent in dispensing other‐regarding duties must prioritise her special relationships’ [17, p. 86]. Molefe is careful to note that partiality does not imply that there are no duties outside of one's special relationships; instead, the idea is that one's duties begin first and foremost with special relationships [17, pp. 86–87].

I am sceptical that such special obligations are enough on their own to justify detaching oneself, *once one has already been attached*. However, reflecting on special obligations admittedly makes matters more complicated. It is not just that your dignity has been violated, but that remaining plugged in will in all likelihood have tangible harms for your community. It is not just the Society of Music Lovers that matters, but also the family and friends of the person who has been plugged in against their will. Here, I admit that intuitions will vary about how to weigh these competing considerations against each other. Still, it is doubtful that special obligations could be weighty enough to justify detaching oneself when the result of doing so is the loss of the human life. Notice, however, that even if I am wrong about this and it would be justified and so yield the intuitive verdict that Thomson is driving, it is for *very different reasons*.

Yet another consideration is that relying on special obligations in this way seems to make the correct verdict relative to one's social relationships. For example, could it be that the hermit who was kidnapped is less justified in unplugging because they are not in special relationships with others who will be harmed by their absence for 9 months? Though I suspect making a person's moral status dependent on community in this way would be considered a bug to many working within the Anglo‐American tradition, it would not surprise me if it were considered a reasonable feature for some working within the African tradition.

### 3.2 The Violinist Case and the Relational Moral Theory

Unlike the view of normative personhood discussed above, analysing the Violinist Case in terms of Metz's Relational Moral Theory is more complex. At first glance, it is tempting to say that the relational moral theory clearly forbids unplugging yourself from the violinist because it would be ‘unfriendly’ (i.e., degrading) to a person who has full moral status in virtue of being able to be both the object and subject of identification and solidarity. It would terminate their life, and so the friendliest (i.e., the most loving) thing you could do is to remain connected to them for 9 months and save their life. In other words, you ought to make the sacrifice for them.

Two further items need to be noted. First, plugging you into the violinist without your consent is an act of unfriendliness. Metz's theory says that it is impermissible to act in unfriendly ways even when doing so promotes friendliness for a greater number of people. His theory has a deontological constraint, which implies that an individual cannot be degraded in this way, regardless of the positive consequences that may follow from it for others [1, p. 114]. It is also permissible to act in unfriendly ways towards aggressors who initiate unfriendly actions against you. Though being plugged into the violinist without your consent is not as degrading as permanently injuring or killing you would be, it is degrading nonetheless. And it would be permissible to use force to prevent yourself from being connected in the first place, though once connected, there do seem to be fairly strong initial reasons to remain plugged in. Second, it needs to be asked how your decision will impact not only the violinist but also the Society of Music Lovers. Despite the fact that rightness as friendliness says that it was wrong to connect you in the first place, once connected, there is some obligation to stay connected.

Here is yet another factor that needs to be considered: Metz has recently suggested ways that the relational moral theory implies the existence of *self‐regarding duties*. Rightness as friendliness implies a duty to be friendly towards oneself where ‘[s]uch a principle would normally prescribe enjoying a sense of togetherness with oneself, acting on a voluntary, trustworthy basis that avoids undermining one's ends, meeting one's own needs, and doing so for one's own sake and out of compassion for oneself’ [29]. On the other hand, it also entails that once should not act in ways that are unfriendly towards oneself, which means not acting in ways that make you feel alienated from yourself, that foster self‐deception or wishful thinking, that make you feel like you cannot depend on yourself, that lack compassion for yourself, and generally from acting in ways that make one's life go worse [29].

Now, it is reasonable to feel humiliated to wake up and find oneself connected to a violinist. Remembering that this case is supposed to be analogous to pregnancy resulting from nonconsensual sex, the point is even more forceful. If sustaining one's health includes mental health and bodily autonomy, then this duty is also violated by remaining plugged in. Finally, there is a sense in which accepting one's circumstances and remaining plugged in accepts the abuse of the situation and does not challenge its exploitative treatment. Furthermore, if you are trapped in bed for 9 months, it will be difficult to act in ways that are friendly to yourself.

By way of reply, I briefly explore two possible responses. First, even if there are self‐regarding duties, it is unclear that a relational property like friendliness can be used to ground them. What does it mean to relate to oneself. Who is the ‘I’ supposed to be in relationship with? Your self, whatever the self amounts to, is identical with your self. Basing self‐regarding duties on a relational capacity makes it sound as if one has to have a fractured personality where one part interacts with another. Second, though special relationships are well‐established in African thought, the same cannot be said of self‐regarding duties, the existence of which remains under suspicion for large portions of the African tradition. Of course, this is not in itself a reason to reject self‐regarding duties, but it does point to the fact that appealing to them will be unpalatable for many working within the African ethical tradition. Finally, notice that even if I am mistaken about this and self‐regarding duties could ground a reason for unplugging oneself from the violinist, such reasons are very different from those in the Anglo‐American tradition. On the latter, it is considerations about preserving one's autonomy, while on the former it is something like a duty to avoid treating oneself discordantly.^8^

However, analysing the Violinist Case in terms of Metz's relational moral theory is more complicated than this initial first pass. Remember that part of the reason why Thomson uses an adult violinist in her example is that she is granting, for the sake of argument the assumption that fetuses have the same moral status as adult humans. But then this makes trying to examine the thought experiment through the lens of the relational moral theory like trying to square a circle. This is because Metz's theory implies a ‘gradualist approach to moral status’ [1, p. 183]. Since gametes, zygotes, embryos, and very early foetuses cannot exhibit identity or solidarity with others, nor do they have aims or intentions with which we could sympathise, ‘if they ever warrant protection, it is only on *indirect grounds*’ [1]. Metz claims that this makes his theory superior to others because it can explain the long‐standing intuition that moral status increases alongside the development of the embryo to infant.^9^ This means ‘that abortion is permissible […] when there is merely an embryo at stake or when the foetus is very young and undeveloped. As a robust communal relation is not possible with these beings, they lack a moral status altogether (or at most have one that is very low), meaning that abortion would not degrade them’ [1, p. 185]. Thus, while the violinist has full moral status on Metz's theory, a foetus does not. The analogy between the violinist and foetus does not hold on the relational moral theory because it takes a gradualist approach to moral status.

To be relevantly analogous to abortion, then, the Violinist Case would have to change such that you wake up connected to an embryo that needs the next 9 months to become viable and survive being disconnected from you. But then according to Metz's theory, at the moment you wake up it is permissible to unplug yourself because the thing from which you unplug does not have any moral status. Though this technically accords with the verdict that Thomson is aiming at, it misses the mark entirely since one of Thomson's main points is that *even if the foetus is a person*, it is still permissible to terminate the pregnancy. There is admittedly much more to say here, but remember that I am not evaluating what the relational moral theory says about abortion in its own right; I am attempting to discover how it would analyse the Violinist Case, and for the reasons mentioned here it turns out to be a muddle, with significant difficulty taking either the thought experiment or the theory on their own terms.^10^

## 4 Tooley's Serum Kitten Case

Another well‐known hypothetical case in the Anglo‐American literature on abortion is Michael Tooley's Kitten Serum Case.^11^ Here is the case in full:

Suppose at some future time a chemical were to be discovered which when injected into the brain of a kitten would cause the kitten to develop into a cat possessing a brain of the sort possessed by humans, and consequently into a cat having all the psychological capabilities characteristic of adult humans. Such cats would be able to think, to use language, and so on. Now it would surely be morally indefensible in such a situation to ascribe a serious right to life to members of the species *Homo sapiens* without also ascribing it to cats that have undergone such a process of development: there would be no morally significant differences [30].

Tooley claims that it would not be wrong to refrain from injecting the newborn kitten with the serum and instead to kill it. Just because it is possible to give the kitten something that would cause it to eventually possess properties that would give it a right to life, does not entail that before that it has a right to life. The fact that it is even possible to initiate such a process does not mean that killing newborn kittens is wrong [30, p. 61]. Likewise, if it is not wrong to fail to initiate such a process, then interfering with that process cannot be wrong either. Tooley argues that this is analogous to the case of taking the life of a foetus that will eventually develop a certain set of properties that will give it a right to life, but that it currently does not possess. The value of a thing is distinct from its potential. Thus, arguments against abortion based on potentiality (i.e., potential for future properties) fail.^12^

### 4.1 The Serum Kitten Case and Normative Personhood

Tooley's thought experiment, if successful, would directly undermine what Molefe says his theory of personhood as sympathy implies about the permissibility of abortion. Consider that he writes that ‘foetuses […] have moral status since they possess the potential to develop the capacity for virtue (sympathy) […] those entities that have the potential for the morally relevant ontological capacity only have partial moral status, which provides a sufficient ground to forbid abortion’.^13^ Thus, Molefe explicitly appeals to their potentiality to ground the moral status of foetuses, the very thing that Tooley wishes to reject. For Molefe, it is the *potential* for the capacity for sympathy that gives the foetus its moral status [4, pp. 87, 90].

I cannot identify anything in the virtue of sympathy that would require one to inject the kitten with a substance that would turn it into an entirely different being, and specifically one that would have the same moral status as humans. If the potential for the kitten to become morally equivalent to humans is analogous to the potential for personhood (i.e., for the capacity for sympathy), then Tooley has raised a serious objection to Molefe's position on abortion.^14^

Where I think Molefe could respond is by challenging the analogy itself, and showing what sympathy requires when it is brought closer to the case of pregnancy. Notice that for both Molefe and Tooley, *nothing* needs to be done to the foetus in order for it to possess the potential for the capacity for sympathy (i.e., personhood). This could be where the analogy between the kitten serum case and pregnancy breaks down. All else being equal, if the embryo or foetus is left alone, then its potential for the capacity for sympathy will eventually become the actual capacity. There is no process that must be initiated to move the embryo from potential to the actual capacity. The process has already started, and its potential is inbuilt. This raises two distinct kinds of potentiality: (i) Potentiality based on a process that needs to be initiated. Suppose the embryo needs to be injected with a serum to become a person with full moral standing. (ii) Potentiality based on a process that is already occurring. Suppose the embryo does not need to be injected with anything and will become a person with full moral standing if left alone, all else being equal. In order for Molefe to reject the analogy, he needs to show that there is a significant moral difference between (i) and (ii). One possible difference is that in the case of (i) you just need to refrain from using the serum and the embryo never becomes a person, whereas in (ii) unless you actively destroy it, it will become a person. Is destroying it less sympathetic than refraining from giving it the serum? This seems like the key point Molefe will have to defend. Of course, proponents of Tooley's argument will note that in (i) and (ii) both have the potential to be the *same* sort of beings, and if their potential is the same, there is no morally relevant difference between the cases. There is more to say here, but again, I am not attempting to adjudicate the disagreement here between Molefe's personhood as sympathy and Tooley's Kitten Serum Case. Rather, I am pointing out the fact that the disagreement itself exists.

### 4.2 The Kitten Serum Case and the Relational Moral Theory

As shown above, Metz claims that his relational moral theory entails that embryos and early‐stage fetuses have no (or very little) moral status. In light of this, if the kitten is supposed to be analogous with a foetus, then there is no obligation to inject it with the serum. Having said that, it is doubtful, or at least unclear, whether it would also be permissible to kill it. Though that kitten (i.e., the foetus) cannot be the *subject* of friendliness, it can be the *object* of it, at least to some extent. For example, Metz assigns partial moral status to animals because they can be the object of identification and solidarity. He writes that ‘In general, the urgent interests of a being with moral status should not be traded off for the trivial interests of another being, even one with a higher moral status’ [1, p. 180]. With respect to the foetus, he writes that ‘once [it] has developed such capacities [i.e., for friendliness], abortion for the sake of realizing the less than urgent interests of the mother is at least pro tanto wrong’ [1, p. 185]. For Metz, it is the very early‐stage embryos that do not have such capacities which means that abortion ‘would not count as very unfriendly or discordant in the way that has been conceived here: there would be no subordination of a being capable of intentional action and no harm done to a being capable of living well’ [1].

Though one probably does not have to inject the kitten with the serum, once the foetus has the capacity to be the object of friendliness, it has some moral status. All of this implies that contra Tooley, it is impermissible to kill the kitten when it is merely an animal since it can be the object of friendliness. In sum, it is doubtful that the verdict Tooley is aiming at can be accepted by the proponent of the relational moral theory, except with respect to early‐stage embryos that can neither be the object nor the subject of friendliness.

## 5 Conclusion

As stated from the outset, it might seem like an uninteresting truism to observe that certain African normative theories—theories often referred to as ‘communitarian’—offer different verdicts on thought experiments from the Anglo‐American tradition. Or, when the verdicts do tend to converge, they do for reasons that are entirely different from the motivations in the Anglo‐American tradition. Taking the time to parse these cases in light of two specific African normative theories puts pressure on any implicit claims these cases might make to objectivity or universalizability.

It might be objected that the analysis I offer misses the point of these examples since they are not intended to be analysed through the lens of any specific normative theory. Rather, the intuitions they purport to generate are intended to be taken on their own terms. If they run counter to the outputs of any normative theory, then this just means they actually offer a defeater for the theory in question. If the African normative theories in question offer guidance that conflicts with the intuitive response to these cases, then this is a strike against their plausibility as theories.

It is admittedly the mark of any good normative theory that it issues the intuitively correct verdicts on a wide range of different ethical scenarios. Nevertheless, if African normative theories do not clearly agree with the intuitions that these Anglo‐American cases are driving at, then this may well be reason to doubt that those same intuitions will be shared across the African continent. It is ultimately an empirical matter, one for sociologists or those doing experimental philosophy, to discover exactly what intuitions these cases generate amongst Black people living across the sub‐Saharan. Since I am not conducting such research, I have instead focused on the fact that African normative theories do not appear to issue the intuitive judgements to these cases that align with the aim of their authors. Though I suspect they do to a significant extent, I do not offer any sociological evidence about the extent to which these normative theories represent the intuitions of indigenous Africans.^15^

Presumably, the authors of these examples intend to generate intuitions about abortion that apply across cultures, even if they never explicitly say as much. My analysis of these cases casts doubt on the extent to which they succeed. As it stands, these cases cannot be said to be offering anything like universal moral truths about the permissibility of abortion. Perhaps the best way to avoid charges of cultural relativism is to give up on the thought experiments and attempt to work out the truth about abortion from agreed upon moral principles, leaving intuitions about thought experiments to fall by the wayside. But this is where the rubber meets the road. Given the significant differences between much of the focus of the Anglo‐American and African normative traditions, I am sceptical that agreement about such principles in the first place could ever be reached.^16^

## Conflicts of Interest

The authors declare no conflicts of interest.

## Data Availability

Data sharing is not applicable to this article as no new data were created or analysed in this study.

## Endnotes

1 Questions about what constitutes African philosophy and the correct methodological approach for conducting it have also been extremely important.

2 Tangwa [18]. He has also stated what he takes to be a ‘moderate’ view with little by way of argument, including any based on the African tradition, in *elements of African bioethics in a western frame* (Langaa Research & Publishing Common Initiative Group, 2010), Chapter 3.

3 Ebeh [21]; Obengo [22]. For a contemporary discussion explicitly appealing to a religious ethic (a rare approach despite the high religiosity on the continent) see Molefe and Maraganedzha [23].

4 Thomson [25, p. 59]. Boonin builds on this argument [26].

5 There are different views about what constitutes the relevant moral community that would take me too far afield to explore here.

6 Indeed, consensus‐based decision‐making at the political level is important in many traditional African societies.

7 Thanks to an anonymous referee for bringing this worry to my attention.

8 Thanks to an anonymous referee for bringing this point to my attention.

9 Metz [1, p. 184]. He provides no sociological evidence that this is in fact a widely‐shared intuition, but I will grant it for the sake of argument.

10 I believe that the considerations about the implications for special obligations and the relational moral theory would be similar to what I discussed above with respect to personhood and so I will not mention them here given space constraints. They probably count for something, but I am doubtful they are weighty enough to justify an action that results in the death of a person (though, again, for Metz, the embryo is not one).

11 At the time of writing this article, it has 1240 citations according to Google Scholar.

12 Tooley [30, p. 61]. Tooley argues that the relevant property that guarantees a human the right to ‘only if it possesses the concept of a self as a continuing subject of experiences and other mental states, and believes that it is itself such a continuing entity’ (p. 57). He also acknowledges that this implies infanticide is permissible. These claims and his defense of them are not relevant for my purposes.

13 Molefe [4, p. 70]. Part of Molefe's own account involves defending a certain interpretation of Menkiti's view of personhood. However, nothing in my analysis rests on agreeing with Molefe's interpretation Menkiti.

14 The only place I can find where Molefe explicitly defends the value of potentiality is by addressing it in the context of a trade‐offs, but the case is question does not require them (see Molefe [4, pp. 91–92].

15 If one wanted to argue that these two thought experiments undermine any African normative theory that suggests a different verdict, you would first have to discover whether they actually generate the relevant intuitions in the culture(s) in question.

16 And of course, agreement in itself is hardly a guarantor of truth.
